# Magnetomechanical Behaviors of Hard-Magnetic Elastomer Membranes Placed in Uniform Magnetic Field

**DOI:** 10.3390/ma17194732

**Published:** 2024-09-26

**Authors:** Wenchao Qu, Jun Chen, Huiming Wang

**Affiliations:** 1 Department of Engineering Mechanics, Zhejiang University, Hangzhou 310027, China; quwenchao@zju.edu.cn (W.Q.); jai@zju.edu.cn (J.C.); 2 Key Laboratory of Soft Machines and Smart Devices of Zhejiang Province, Zhejiang University, Hangzhou 310027, China

**Keywords:** hard-magnetic elastomer membrane, large deformation, viscoelastic behavior, instability, magnetic field

## Abstract

This paper aims to develop a theoretical model for a viscoelastic hard-magnetic elastomer membrane (HMEM) actuated by pressure and uniform magnetic field. The HMEM is initially a flat, circular film with a fixed boundary. The HMEM undergoes nonlinear large deformations in the transverse direction. The viscoelastic behaviors are characterized by using a rheological model composed of a spring in parallel with a Maxwell unit. The governing equations for magneto-visco-hyperelastic membrane under the axisymmetric large deformation are constructed. The Zeeman energy, which is related to the magnetization of the HMEM and the magnetic flux density, is employed. The governing equations are solved by the shooting method and the improved Euler method. Several numerical examples are implemented by varying the magnitude of the pre-stretch, pressure, and applied magnetic field. Under different magnetic fields, field variables such as latitudinal stress exhibit distinct curves in the radial direction. It is observed that these varying curves intersect at a point. The position of the intersection point is independent of the applied magnetic field and only controlled by pressure and pre-stretch. On the left side of the intersection point, the field variables increase as magnetic field strength increases. However, on the other side, this trend is reversed. During viscoelastic evolution, one can find that the magnetic field can be used to modulate the instability behaviors of the HMEM. These findings may provide valuable insights into the design of the hard-magnetic elastomer membrane structures and actuators.

## 1. Introduction

Magnetoelastic polymers can alter their mechanical properties and undergo reversible nonlinear large deformation in the presence of an external magnetic field; hence, they are known as smart composite materials. The manufacturing method involves dispersing micron-sized magnetizable particles [1] or embedding discrete magnets [2] in polymers and then solidifying the mixture [3]. The magnetizable particles possess magnetization after being magnetized. Though the polymer matrix does not possess residual magnetic flux density, the magnetoelastic polymers composed of magnetizable particles embedded in the polymer matrix exhibit residual magnetic flux density [4]. When magnetoelastic polymers are placed in an external magnetic field, the mechanical properties can be altered [5]. Additionally, magnetic fields, as external stimuli, possess excellent characteristics such as rapid response [6], long-distance controllability [7], ease of manipulation in enclosed spaces [8,9,10], etc. These eminent characteristics have been utilized in engineering applications and biomedical devices, including flexible robotics [11,12,13], actuators [14,15], biomedicine [16,17,18], and vibration isolation [19,20,21]. Generally, magnetic particles can be classified into two categories: hard-magnetic particles (e.g., neodymium-iron-boron) and soft-magnetic particles (e.g., Fe_3_O_4_). Hard-magnetic particles have a high coercivity and thus possess the ability to maintain their residual magnetization strength unchanged in the absence/presence of an external magnetic field. Polymers with added hard-magnetic particles are named as hard-magnetic materials [22,23].

The theoretical research on magnetoelasticmaterials can be traced back to the 1950s–1960s. The theoretical formulation of the magnetoelasticity for magnetoelastic materials can be approached by using two categories of methods. One is based on the conservation laws of continuum mechanics, also known as the direct method. For instance, Truesdell and Toupin [24], Tiersten [25], Maugin and Erignen [26], and Pao [27] treated the magnetoelastic materials as continua and derived the governing equations through the balance laws. The other is the energy approach, which involves the variational method to derive the governing equations, such as the works by Tiersten [28] and Brown [29]. With the improvement in computational capabilities and the rising applications of magnetoelastic materials in engineering, the modeling and numerical analysis of magnetoelastic structures have captured the attention of researchers. Dorfmann and Ogden [30,31], Kankanala and Triantafyllidis [32], and Steigmann [33] developed a comprehensively nonlinear magnetoelastic field theory to construct the interactions between the external magnetic field and isotropic deformable continua by treating the magnetic field or the magnetic induction as the independent variables based on the variational form of the energy method. The expression of the total free energy density consists of the deformation gradient and magnetic variable (one of the magnetic flux density, field intensity, and magnetization vector). Saxena et al. [34] and Haldar et al. [35] developed a finite deformation magneto-viscoelastic theory to examine the case with energy dissipation. With the consideration of the anisotropy, Bustamante [36] solved the boundary-value problems involving the extension, inflation, and torsion of a transversely isotropic circular cylinder. Danas et al. [5] studied the mechanical response of magnetoelastic materials by curing the samples under the external magnetic field, allowing homogenous micro-sized magnetic particles to rearrange into a chain-like structure, thereby forming transversely isotropic materials. Saxena et al. [37,38] analyzed the mechanical behavior of transverse isotropic magnetoelastic materials through theoretical modeling and finite element simulation. Due to the emergence of hard-magnetic soft materials, the relevant studies are also advancing constantly. Lum et al. [39] established a pioneer work involving theoretical modeling, numerical calculation, and fabrication techniques for designing magnetic soft robotics. Hu et al. [11] created an untethered small-scale robot capable of multimodal locomotion. Using 3D printing technology, Kim et al. [23] successfully created diverse, flexible robots by embedding the hard-magnetic particles into the soft matrix. These robots, driven by a magnetic field, can perform actions like jumping, rolling, and rotating. Kim et al. [40] fabricated a small soft continuum robot that could be actuated by remote magnets. They also analyzed the influence of hard-magnetic particle volume fraction on the shear modulus. Significant achievements have also been gained in theoretical modeling of hard-magnetic materials. Zhao et al. [22] proposed a magnetic potential energy density function based on the assumptions of the ideal hard-magnetic soft materials. By comparing the simulation results by commercial finite-element software ABAQUS (v2016) with experimental data, the nonlinear field theory derived from the continuum mechanics framework is validated. Garcia-Gonzalez [41] constructed a mathematical modeling of the magneto-viscoelasticity of hard-magnetic materials and implemented simulation in a finite element (FE) framework. Chen et al. [4,42,43,44,45] established the mathematical modeling and explored the static and dynamic behavior of the hard-magnetic soft beam. Besides, membrane structures are ubiquitous in nature and have found extensive applications in engineering structures and the aerospace industry [46,47]. Duffett and Reddy [48] and Gruttmann and Taylor [49] proposed a finite formulation for describing the large deformation of membranes, including plane stress and axisymmetric problems. Neto et al. [50] described a finite element formulation of finite hyperelasticity setting on spatial configuration. Steigmann [51] derived a concise membrane theory from 3D elasticity theory based on asymptotic or variational methods. Kanner and Horgan [52] investigated the effects of strain-stiffening on the classical limit point instability during internal pressure inflation of rubber-like spherical and cylindrical thin-walled shells made of incompressible isotropic materials. Il’ichev and Fu [53] studied the stability of locally wall-thinned inflated hyperelastic membrane tubes. Steigmann [33] extended the framework of magnetoelastic theory to magnetoelastic membranes using formulations based on magnetic field intensity. Barham et al. [54,55,56,57] studied the critical point instability of magnetoelastic membranes and the mechanical response of a weakly magnetized circular membrane under a stationary dipole. The wrinkling and instabilities in pressurized toroidal and cylindrical magnetoelastic membranes were systematically analyzed by Reddy and Saxena [58,59] and Saxena et al. [60]. Ali et al. [61] developed a 2D-magnetoelastic membrane theory placed in an azimuthal magnetic field with a weakly magnetizable material and numerical examples are implemented. In summary, there is a body of literature that describes the mechanical behaviors of magnetoelastic materials, which are composed of soft-magnetic particles.

However, the investigations on the HMEM are relatively limited. Hard-magnetic elastomer membranes, as an emerging smart composite material, are capable of high coercivity and can provide new avenues for engineering applications, such as bacterial cellulose [62] and actuators [63]. Due to the strong nonlinearity and magneto-mechanical coupling effect, the modeling and analysis of the deformation behavior of hard-magnetic soft membranes is still a challenge. Thus, it is vital to theoretically study the magnetomechanical behaviors of the HMEM subject to coupled magneto-pneumatic loadings. Herein, we present a theoretical model for hard-magnetic elastomer membranes to study the magnetomechanical deformations of the HMEM by employing Zeeman magnetic energy [64]. Additionally, viscoelasticity, as an intrinsic characteristic of soft materials, is considered by introducing a rheological model. To reveal the complex deformation mechanisms, two mechanical loading modes are considered: (i) The hyperelastic and viscoelastic deformations of the HMEM are studied under pure pressure. (ii) The magnetomechanical behaviors of the HMEM under pre-stretch and magneto-pneumatic coupling are further investigated. The magneto-visco-hyperelastic deformations are systematically analyzed by implementing several numerical cases. This study is organized as follows: The basic equations of nonlinear magnetoelasticity are presented in Section 2. The theoretical model for hard-magnetic elastomer membranes is developed in Section 3. The numerical results for the illustrative examples are discussed in Section 4. Finally, the concluding statements for the magnetomechanical behaviors of the HMEM concerning magneto-pneumatic coupling effects are drawn.

## 2. Basic Equations of Nonlinear Magnetoelasticity

The kinematics of the magnetoelastic continua in the continuum mechanics framework can be seen in the works by Ali et al. [61] and Dorfmann and Ogden [64]. 

The Cauchy stress tensor σ is represented by

(1) $$\sigma =J^{-1}PF^{T}$$

where *J* is the determinant of **F**. 

The first Piola-Kirchhoff **P** stress tensor can be written as

(2) $$P=J\sigma F^{-T}$$

In the current configuration, the Eulerian magnetic field vector **H** and Eulerian magnetic induction vector **B** are connected by the Eulerian magnetization such that

(3) $$B=\mu_{0}(H+M)$$

where $\mu_{0}$ represents the vacuum permeability.

For an incompressible material, the total nominal stress tensor in Equation (2) can be rewritten as

(4) $$P=\frac{\partial \tilde{W}}{\partial F}-qF^{-T}$$

where *q* is the Lagrange multiplier.

The total free energy of the hard-magnetic soft materials composed of an elastic part and a magnetic part is expressed as

(5) $$\tilde{W}={\tilde{W}}^{elastic}+{\tilde{W}}^{magnetic}$$

## 3. Hard-Magnetic Elastomer Membrane Theory

### 3.1. Nonlinear Magnetoelasticity

#### 3.1.1. Deformation Procedures of the HMEM

**Figure 1:**
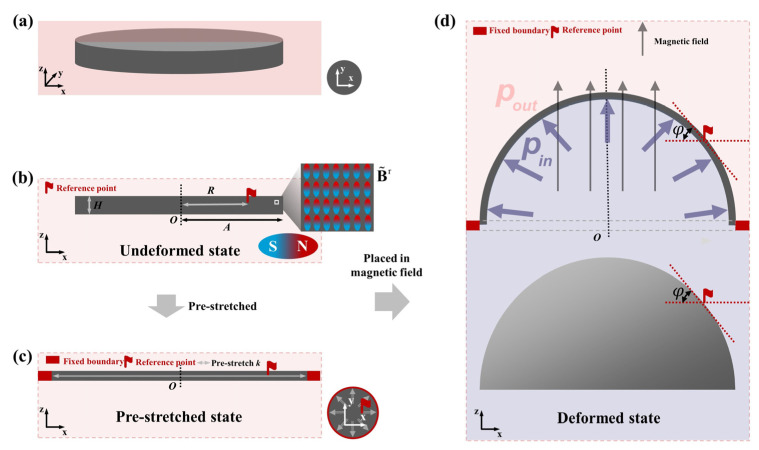
Mechanical model of the HMEM: (**a**) The unmagnetized state. (**b**) The undeformed state. (**c**) The pre-stretched state. (**d**) The deformed state. ${\tilde{B}}^{r}$ denotes the residual magnetic flux density of the HMEM. The pressure *p* ($p_{in}-p_{out}$) is set as the constant value. *k* is the pre-stretch.

The deformation procedures of the HMEM are as follows: (i) Consider an unmagnetized membrane in Figure 1a. (ii) Then, the hard-magnetic particles are magnetically saturated in Figure 1b, indicating that the HMEM possesses residual magnetic flux density ${\tilde{B}}^{r}$ along with the *z*-axis direction. In the undeformed state, the HMEM is of a flat circular membrane without restriction. The geometrical parameters, such as radius and thickness, are set as $R_{0}$ and *H*, respectively. *o* is the origin of the coordinate system. (iii) Subsequently, the HMEM mounting on a rigid ring is homogeneously pre-stretched to radius *a*, as shown in Figure 1c. (iv) The HMEM is inflated by a constant pressure *p* and actuated by uniform magnetic fields $B^{applied}$ parallel with the *z*-axis in Figure 1d.

#### 3.1.2. Kinematics

**Figure 2:**
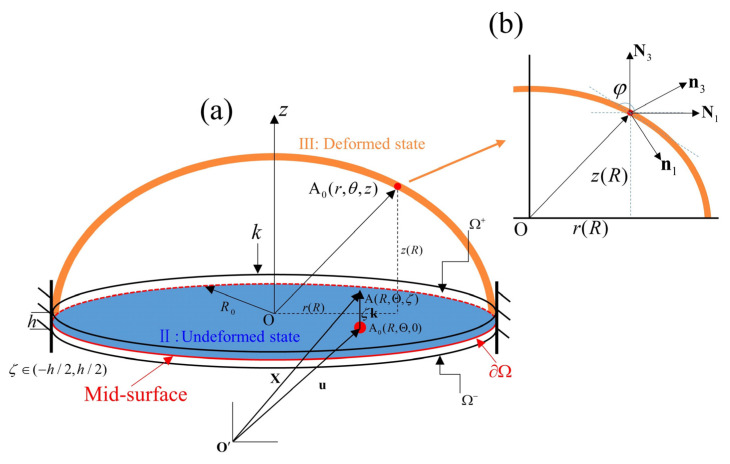
(**a**) The kinematics of the HMEM under II: the pre-stretched state and III: the deformed state. (**b**) The axisymmetric deformation of the HMEM. *φ* denotes the angle between the basis vector $N_{1}$ and the tangent of the material point (red dot) of the HMEM.

The position vector of the material point A (R,Θ,ζ) can be represented as

(6) $$X=RE_{R}(\Theta )+\zeta k$$

where $R\in [0,R_{0}]$, Θ, and ζ∈[−H/2,H/2] represent cylindrical coordinates in the reference configuration, as illustrated in Figure 2. $E_{R}$, $E_{\Theta }$, and k represent unit vectors in radial, azimuthal, and axial directions in the reference configuration, respectively. The position vector of the material point A in the deformed state can be expressed as

(7) $$x(R,\;\Theta ,\;\zeta )=r(R,\Theta ,\zeta )e_{r}(\theta (R,\Theta ,\zeta ))+z(R,\Theta ,\zeta )k$$

where *r*, θ, and z are the corresponding cylindrical coordinates in the current configuration. $e_{r}$, $e_{\theta }$, and **k** are the basis vectors in radial, azimuthal, and axial directions in the reference configuration, respectively.

For the axisymmetric deformation, we have θ=Θ. Thus, the basis vector in the reference configuration is consistent with those in the deformed configuration, i.e., $e_{\theta }=E_{\Theta }$ and $e_{r}=E_{R}$. Equation (7) can be rewritten as

(8) $$x(R,\;\zeta )=r(R,\zeta )e_{r}(\Theta )+z(R,\zeta )k$$

The deformation gradient **F** can be written as

(9) $$F=\frac{\partial x}{\partial X}=(\frac{\partial r}{\partial R}e_{r}+\frac{\partial z}{\partial R}k)⊗E_{R}+\frac{r}{R}e_{\theta }⊗E_{\Theta }+(\frac{\partial r}{\partial \zeta }e_{r}+\frac{\partial z}{\partial \zeta }k)⊗k$$

The right Cauchy-Green deformation tensor **C** is expressed as

(10) $$\begin{array}{l} C=F^{T}F=\left[{\left(\frac{\partial r}{\partial R}\right)}^{2}+{\left(\frac{\partial z}{\partial R}\right)}^{2}\right]E_{R}⊗E_{R}+{\left(\frac{r}{R}\right)}^{2}E_{\Theta }⊗E_{\Theta }+\left[{\left(r^{\prime }\right)}^{2}+{\left(z^{\prime }\right)}^{2}\right]k⊗k\\ \;\;+(\frac{\partial r}{\partial \zeta }\frac{\partial r}{\partial R}+\frac{\partial z}{\partial \zeta }\frac{\partial z}{\partial R})\left[E_{R}⊗k+k⊗E_{R}\right] \end{array}$$

The neo-Hookean model is considered, and the Zeeman energy per unit reference volume [64] is adopted for the total energy. Therefore, Equation (5) can be rewritten as

(11) $${\tilde{W}}^{\mathrm{magnetic}}=\frac{G}{2}(I_{1}-3)-M_{L}\cdot B_{L}+\frac{1}{2}{\left(\mu_{0}J\right)}^{-1}B_{L}\cdot (CB_{L})$$

where *G* denotes the shear modulus and $I_{1}=\mathrm{tr}(C)=\lambda_{1}^{2}+\lambda_{2}^{2}+\lambda_{3}^{2}$. $M_{L}$ and $B_{L}$ represent the Lagrangian magnetization and Lagrangian magnetic induction, respectively.

$\lambda_{i}(i=1,2,3)$ denotes the principle stretches, i.e., the eigenvalues of the left and right stretch tensors $U=\sqrt{F^{T}F}$ and $V=\sqrt{FF^{T}}$. By introducing the orthonormal base vector $\left(N_{1},N_{2},N_{3}\right)$ and $\left(n_{1},n_{2},n_{3}\right)$, the stretch tensors are obtained as

(12) $$U=\sum_{i=1}^{3}\lambda_{i}N_{i}⊗N_{i},\;V=\sum_{i=1}^{3}\lambda_{i}n_{i}⊗n_{i}$$

and the deformation gradient tensor is expressed as

(13) $$F=\sum_{i=1}^{3}\lambda_{i}n_{i}⊗N_{i}$$

where $n_{i}=RN_{i}$, and **R** represents the orthogonal rotation tensor associated with the polar decomposition of **F**, expressed as **F** = **RU** = **VR**.

The rotation tensor **R** can be given by

(14) $$R=\left[\begin{array}{ccc} \cos\varphi & 0 & \sin\varphi \\ 0 & 1 & 0\\ -\sin\varphi & 0 & \cos\varphi \end{array}\right]$$

where φ represents the angle between the horizontal plane and the tangential direction.

The first Piola-Kirchhoff **P** stress tensor in Equation (4) is rewritten as

(15) $$P=\sum_{i=1}^{3}(\frac{\partial \tilde{W}}{\partial \lambda_{i}}-\frac{q}{\lambda_{i}})n_{i}⊗N_{i}\;(q=\lambda_{3}\frac{\partial \tilde{W}}{\partial \lambda_{3}})$$

By definition, the right Cauchy-Green deformation tensor **C** is diagonal and $E_{R}$, $E_{\Theta }$, and **k** are the principal axes; thus, we have

(16) $$N_{1}=E_{R},\;N_{2}=E_{\Theta },\;N_{3}=k$$

By substituting Equation (16) into Equations (9) and (13), one can obtain

(17) $$n_{1}=\frac{1}{\lambda_{1}}(\frac{\partial r}{\partial R}e_{r}+\frac{\partial z}{\partial R}k),\;n_{2}=e_{\theta },\;n=\frac{1}{\lambda_{3}}(\frac{\partial r}{\partial \zeta }e_{r}+\frac{\partial z}{\partial \zeta }k)$$

The substitution of Equation (16), $e_{\theta }=E_{\Theta }$, and $e_{r}=E_{R}$ into Equation (17) yields

(18) $$\cos\varphi =\frac{1}{\lambda_{1}}\frac{\partial r}{\partial R}=\frac{\partial z}{\partial \zeta }\frac{1}{\lambda_{3}},\;\sin\varphi =-\frac{1}{\lambda_{1}}\frac{\partial z}{\partial R}=\frac{\partial r}{\partial \zeta }\frac{1}{\lambda_{3}}$$

The principal stretches can be obtained from Equation (10) as

(19) $$\lambda_{1}={\left[{\left(\frac{\partial r}{\partial R}\right)}^{2}+{\left(\frac{\partial z}{\partial R}\right)}^{2}\right]}^{1/2},\;\lambda_{2}=\frac{r}{R},\;\lambda_{3}={\left[{\left(\frac{\partial r}{\partial \zeta }\right)}^{2}+{\left(\frac{\partial z}{\partial \zeta }\right)}^{2}\right]}^{1/2}$$

The mechanical equilibrium equations [61] can be expressed as

(20) $$\frac{\partial P_{rR}}{\partial R}+\frac{1}{R}(P_{rR}-P_{\theta \Theta })=P\frac{\partial z}{\partial R}\frac{r}{R}$$

(21) $$\frac{\partial P_{zR}}{\partial R}+\frac{1}{R}P_{zR}=-P\frac{\partial r}{\partial R}\frac{r}{R}$$

The components of the first Piola-Kirchhoff stress are obtained using Equations (15) and (17) as

(22) $$P_{rR}=s_{1}\cos\varphi ,\;P_{zR}=-s_{1}\sin\varphi ,\;P_{\theta \Theta }=s_{2}$$

By substituting Equation (22) into Equations (20) and (21), we can obtain

(23) $$\frac{ds_{1}}{dR}+\frac{1}{R}(s_{1}-s_{2}\cos\varphi )=0$$

(24) $$s_{1}\frac{d\varphi }{dR}+\frac{1}{R}s_{2}\cos\varphi =\lambda_{1}\lambda_{2}p$$

where $s_{1}$ and $s_{2}$ are the principal stress along the principal stretches $\lambda_{1}$ and $\lambda_{2}$, respectively. By taking the derivative of $\lambda_{2}$, the geometric relationship is given by

(25) $$\frac{d\lambda_{2}}{dR}=\frac{1}{R}(\lambda_{1}\cos\varphi -\lambda_{2})$$

Therefore, φ, $\lambda_{1}$, $\lambda_{2}$, and z are four unknown variables to be solved.

Due to the restriction of the boundary conditions, we can obtain

(26) $$r(R_{0},t)=a,z(R_{0},t)=0.$$

(27) φ(0,t)=0,r(0,t)=0.

where *t* represents the time variable.

#### 3.1.3. Viscoelasticity

**Figure 3:**
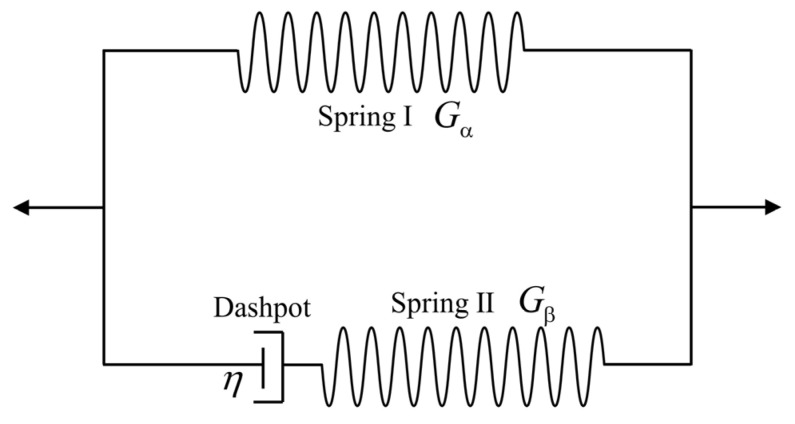
The rheological model is composed of a spring in parallel with a Maxwell unit. $G_{\alpha }$ and $G_{\beta }$ are set as the shear modulus of the spring Ⅰ and II, respectively.

Here, a rheological model [65] is utilized to characterize the viscoelastic behavior. The model is composed of a pure spring and a Maxwell unit, as shown in Figure 3. $G_{\alpha }$ and $G_{\beta }$ denotes the shear modulus of the springs Ⅰ and II, respectively. η denotes the viscosity coefficient of the dashpot.

Thus, we can obtain the stretches of the spring II

(28) $$\lambda_{1}^{e}=\lambda_{1}\xi_{1}^{-1}$$

(29) $$\lambda_{2}^{e}=\lambda_{2}\xi_{2}^{-1}$$

where the stretches of the dashpot are denoted by the internal variables $\xi_{1}$ and $\xi_{2}$.

The substitution of Equations (28) and (29) into Equation (11) yields

(30) $$\begin{array}{l} \tilde{W}=\frac{G_{\alpha }}{2}(\lambda_{1}^{2}+\lambda_{2}^{2}+\lambda_{1}^{-2}\lambda_{2}^{-2}-3)+\frac{G_{\beta }}{2}(\lambda_{1}^{2}\xi_{1}^{-2}+\lambda_{2}^{2}\xi_{2}^{-2}+\lambda_{1}^{-2}\lambda_{2}^{-2}\xi_{1}^{2}\xi_{2}^{2}-3)\\ \;\;-M_{3}\cdot B_{3}+\frac{1}{2}{\left(\mu_{0}J\right)}^{-1}\lambda_{1}^{-2}\lambda_{2}^{-2}B_{3}^{2} \end{array}$$

where subscript ‘3′ represents the *z*-axis direction.

Based on the non-equilibrium thermodynamic theory [66], the evolution equations can be defined as

(31) $$\frac{1}{\xi_{1}}\frac{d\xi_{1}}{dt}=\frac{1}{3\eta }\left[G_{\beta }(\lambda_{1}^{2}\xi_{1}^{-2}-\xi_{1}^{2}\xi_{2}^{2}\lambda_{1}^{-2}\lambda_{2}^{-2})-\frac{G_{\beta }}{2}(\lambda_{2}^{2}\xi_{2}^{-2}-\xi_{1}^{2}\xi_{2}^{2}\lambda_{1}^{-2}\lambda_{2}^{-2})\right]$$

(32) $$\frac{1}{\xi_{2}}\frac{d\xi_{2}}{dt}=\frac{1}{3\eta }\left[G_{\beta }(\lambda_{2}^{2}\xi_{2}^{-2}-\xi_{1}^{2}\xi_{2}^{2}\lambda_{1}^{-2}\lambda_{2}^{-2})-\frac{G_{\beta }}{2}(\lambda_{1}^{2}\xi_{1}^{-2}-\xi_{1}^{2}\xi_{2}^{2}\lambda_{1}^{-2}\lambda_{2}^{-2})\right]$$

Treating the viscosity of the dashpot as an infinite and setting $\xi_{1}$ = $\xi_{2}$ = 1 in Equations (28), (29), (31), and (32) results in the HMEM exhibiting hyperelastic deformation. Suppose the pre-stretch process is accomplished in a short time, and the creep in this period is ignored. The initial conditions are given by

(33) $$\xi_{1}(R,0)=1,\;\;\xi_{2}(R,0)=1$$

The nominal stress $s_{1}$ and $s_{2}$ are obtained as

(34) $$s_{1}=\frac{\partial \tilde{W}}{\partial \lambda_{1}}=G_{\alpha }(\lambda_{1}-\lambda_{1}^{-3}\lambda_{2}^{-2})+G_{\beta }(\lambda_{1}\xi_{1}^{-2}-\xi_{1}^{2}\xi_{2}^{2}\lambda_{1}^{-3}\lambda_{2}^{-2})-\frac{\lambda_{1}^{-3}\lambda_{2}^{-2}}{\mu_{0}}B_{3}^{2}$$

(35) $$s_{2}=\frac{\partial \tilde{W}}{\partial \lambda_{2}}=G_{\alpha }(\lambda_{2}-\lambda_{1}^{-2}\lambda_{2}^{-3})+G_{\beta }(\lambda_{2}\xi_{2}^{-2}-\xi_{1}^{2}\xi_{2}^{2}\lambda_{1}^{-2}\lambda_{2}^{-3})-\frac{\lambda_{1}^{-2}\lambda_{2}^{-3}}{\mu_{0}}B_{3}^{2}$$

Taking the derivative of Equation (34) yields $ds_{1}/dR$. And then substituting Equation (23) into $ds_{1}/dR$, one can obtain

(36) $$\frac{d\lambda_{1}}{dR}=\frac{1}{f_{1}}\left[(\frac{s_{2}cos\varphi -s_{1}}{R})-f_{2}(\frac{\lambda_{1}cos\varphi -\lambda_{2}}{R})+f_{3}\frac{d\xi_{1}}{dR}+f_{4}\frac{d\xi_{2}}{dR}\right]$$

where

(37) $$\begin{array}{l} f_{1}=G_{\alpha }+3G_{\alpha }\lambda_{1}^{-4}\lambda_{2}^{-2}+G_{\beta }\xi_{1}^{-2}+3G_{\beta }\xi_{1}^{2}\xi_{2}^{2}\lambda_{1}^{-4}\lambda_{2}^{-2}+3\lambda_{2}^{-2}\lambda_{1}^{-4}B_{3}^{2}/\mu_{0}\\ f_{2}=2G_{\alpha }\lambda_{1}^{-3}\lambda_{2}^{-3}+2G_{\beta }\xi_{1}^{2}\xi_{2}^{2}\lambda_{1}^{-3}\lambda_{2}^{-3}+2\lambda_{1}^{-3}\lambda_{2}^{-3}B_{3}^{2}/\mu_{0}\\ f_{3}=2G_{\beta }(\lambda_{1}^{-3}\lambda_{2}^{-2}\xi_{1}\xi_{2}^{2}+\lambda_{1}\xi_{1}^{-3})\\ f_{4}=2G_{\beta }\lambda_{1}^{-3}\lambda_{2}^{-2}\xi_{1}^{2}\xi_{2} \end{array}$$

The non-dimensional governing equations are listed in the Appendix A.

### 3.2. Solving Procedure

**Figure 4:**
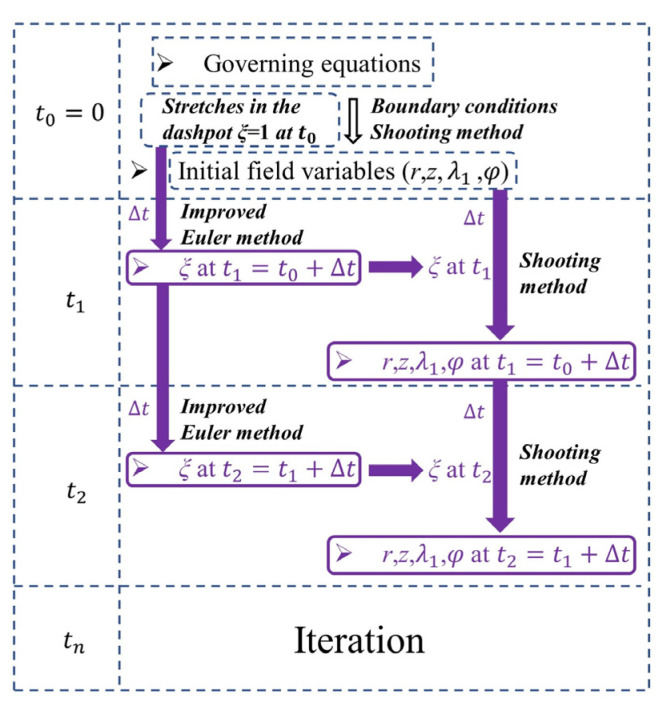
The solving procedure of the field variables.

**Figure 5:**
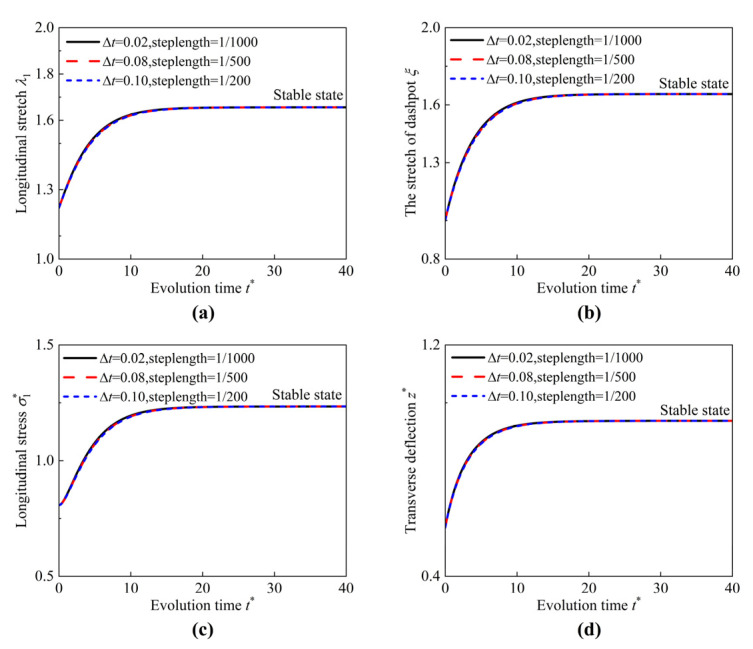
Evolution of (**a**) longitudinal stretch $\lambda_{1}$, (**b**) the stretch of dashpot ξ, (**c**) longitudinal stress $\sigma_{1}^{*}$, and (**d**) transverse deflection $z^{*}$ under different time steps and discretization step length.

Here, the notes on solving techniques are provided. The field variables r(R,t), $\lambda_{1}(R,t)$, φ(R,t), and z(R,t) have been given in Equations (A1)–(A8) in the Appendix A. The internal variables $\xi_{1}(R,t)$ and $\xi_{2}(R,t)$ are the functions that are dependent on time *t*. According to the initial conditions Equation (33), all the physical fields r(R,t), $\lambda_{1}(R,t)$, φ(R,t), and z(R,t) can be determined by using the shooting method when time *t* = 0, as shown in Figure 4. The ODE45 in MATLAB is used to implement the calculations. Subsequently, by choosing an appropriate time step Δt, the stretches of the dashpot $\xi_{1}$ and $\xi_{2}$ at the next time step $t_{1}=t_{0}+\Delta t$ can be calculated from Equations (31) and (32) by using the improved Euler method. Then, at the present time $t_{1}=t_{0}+\Delta t$, all the physical fields can be obtained by using the shooting method again. Finally, by repeatedly performing this process, the governing equations can be solved, and all the physical fields can be obtained step by step. By choosing different time steps and discretization step length in the radial direction, the stability and convergence of the shooting method and the improved Euler method are demonstrated, as illustrated in Figure 5.

## 4. Results and Discussions

### 4.1. Validation

**Figure 6:**
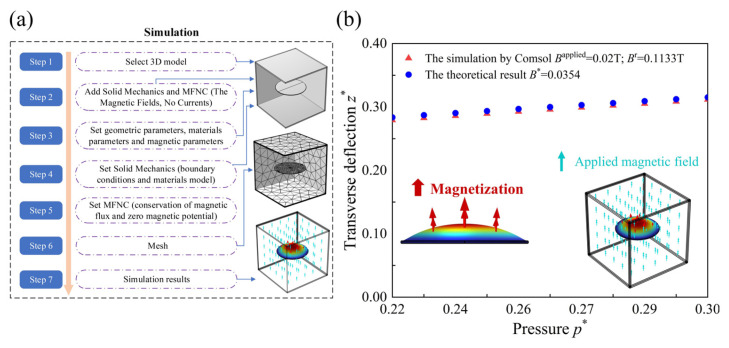
(a) The simulation procedure by COMSOL. (b) The comparison between the current results and simulation by COMSOL.

The validation of the current numerical results is conducted by comparing them with the finite element result obtained from COMSOL (the combination of MFNC (The Magnetic Fields, No Currents) and SOLID (The Solid Mechanics) interfaces). The corresponding simulation procedure is illustrated in Figure 6a. $B^{r}$ and $B^{applied}$ are the actual residual magnetic flux density and applied magnetic flux density, respectively. Given the absence of bending stiffness in the HMEM, the radius $R_{0}$ of the HMEM significantly exceeds its thickness *H*. As the pressure $p^{*}$ increases from 0.215 to 0.300, the transverse deflection $z^{*}$ (red triangles) by COMSOL gradually enhances under $B^{r}$ = 0.1133 T and $B^{applied}$ = 0.02 T, as shown in Figure 6b. Based on the same magnetic loading parameters (dimensionless form $B^{*}$ = 0.0354), the current numerical results (blue dot) show good agreement with the simulation results by COMSOL.

### 4.2. Magnetomechanical Behaviors under Magneto-Pneumatic Coupling

**Figure 7:**
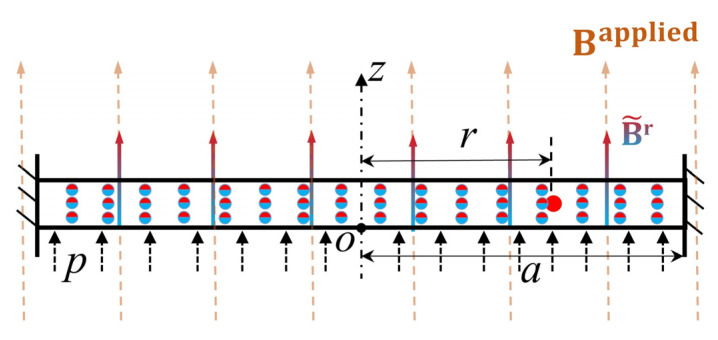
The HMEM in the undeformed state.

In this subsection, the viscoelastic deformation of the HMEM under magneto-pneumatic coupling will be discussed. To simplify the calculations, the shear modulus of the spring Ⅰ and II are set as $G_{\alpha }=G_{\beta }=G/2$. The viscoelastic relaxation time is set as $t_{v}=\eta /G_{\beta }$. The HMEM is placed in a uniform magnetic field, as shown in Figure 7. The direction of the magnetic field strength and residual magnetic flux density are both parallel to the *z*-axis.

**Figure 8:**
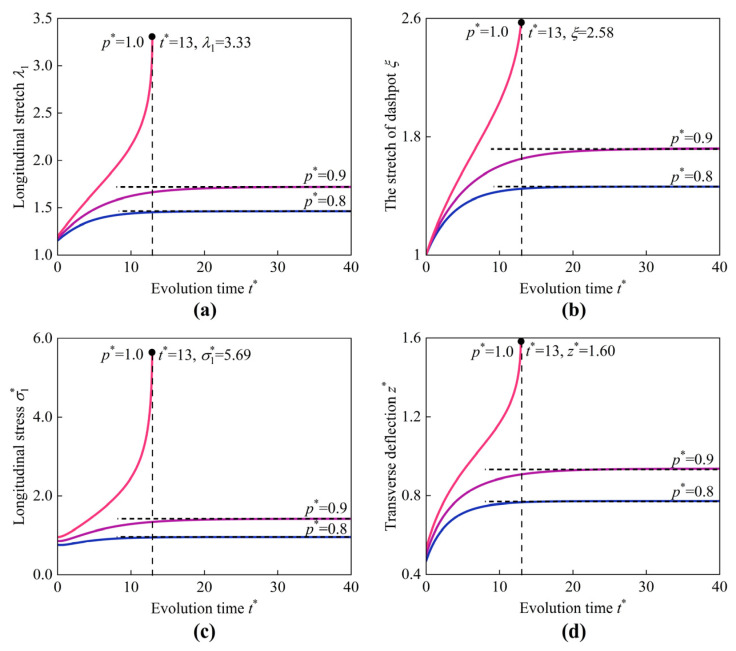
Evolution of (**a**) longitudinal stretch $\lambda_{1}$, (**b**) the stretch of dashpot ξ, (**c**) longitudinal stress $\sigma_{1}^{*}$, and (**d**) transverse deflection $z^{*}$ at the center of the HMEM under different values of $p^{*}$ ($B^{applied}$ = 0).

First, we consider the viscoelastic deformations of the HMEM under pure pressure (in the absence of applied magnetic fields). When inflated by pure pressure, the HMEM experiences viscoelastic deformation as depicted in Figure 8a–d, illustrating the evolution of the longitudinal stretch $\lambda_{1}$, the stretch of the dashpot ξ, the longitudinal stress $\sigma_{1}^{*}$, and the transverse deflection $z^{*}$ at the center of the HMEM, respectively. At the first stage, it is supposed that the HMEM is inflated by pressure within a very short time, and the stretch of the dashpot ξ is assumed to remain unchanged, say ξ = 1. At this stage, the elastic deformation of the HMEM is carried by spring Ⅰ and II together. As the dashpot is fully relaxed, the stress and stretch in the spring II gradually decreases until it reaches zero. All the physics quantities, including $\lambda_{1}$, ξ, $\sigma_{1}^{*}$, and $z^{*}$ eventually evolve into constant values, as represented by the dark horizontal dash-dot lines, indicating the equilibrium state. For instance, when $t^{*}$ surpasses 20, the variations in $\lambda_{1}$ are very small under $p^{*}$ = 0.8 and 0.9, respectively. However, when $p^{*}$ increases to 1.0, the HMEM evolves from a stable state into an unstable state. The HMEM cannot reach the mechanical equilibrium when $t^{*}>$ 13 under $p^{*}$ = 1.0. An instability occurs in the range $p^{*}$ = 0.9 to 1.0.

**Figure 9:**
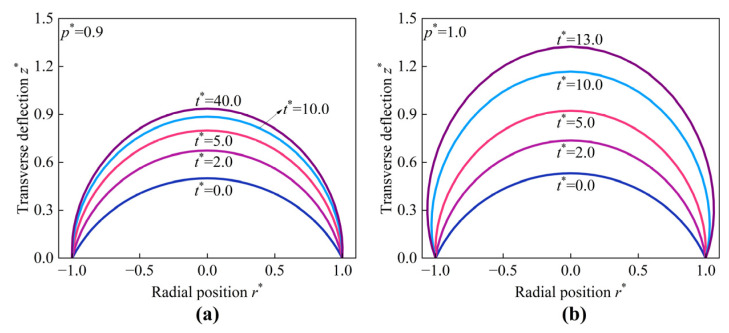
The evolution shape of the HMEM under (**a**) $p^{*}$ = 0.9 and (**b**) $p^{*}$ = 1.0.

The actual deformation shapes of the HMEM under $p^{*}$ = 0.9 and $p^{*}$ = 1.0 at different evolution times $t^{*}$ are depicted in Figure 9. For $p^{*}$ = 0.9, the variations in the transverse deflection $z^{*}$ decreases gradually as evolution time $t^{*}$ increases. However, when $p^{*}$ = 1.0, the transverse deflection $z^{*}$ increases dramatically, leading to instability or fracture.

**Figure 10:**
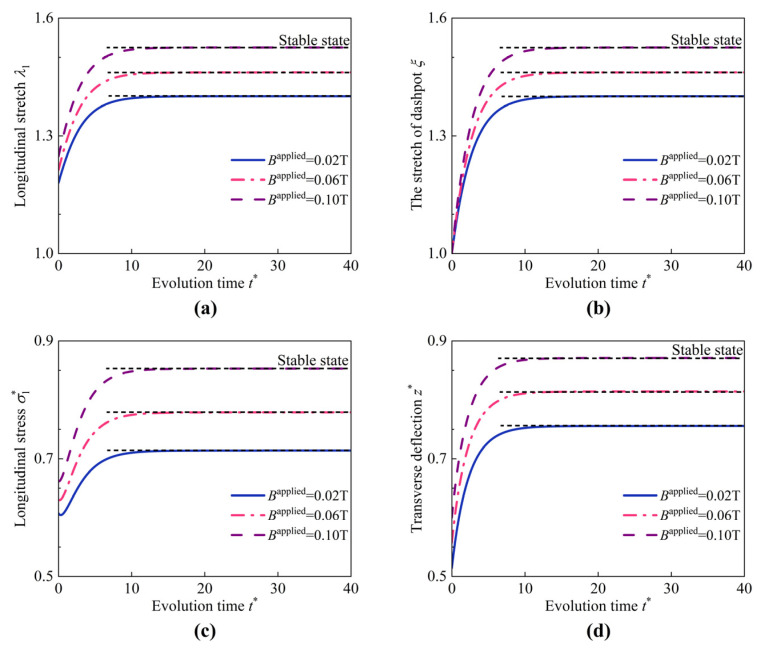
Evolution of (**a**) longitudinal stretch $\lambda_{1}$, (**b**) the stretch of dashpot ξ, (**c**) longitudinal stress $\sigma_{1}^{*}$, and (**d**) transverse deflection $z^{*}$ at the center of the HMEM under different values of $B^{applied}$ ($p^{*}$ = 0.6).

Next, the magnetomechanical behaviors of the HMEM under magneto-pneumatic coupling are investigated. The loading parameters of the magneto-pneumatic coupling studied here are expressed as ($p^{*}$, $B^{applied}$). The $B^{applied}$ ranges from 0.02 T to 0.1 T with an interval of 0.04 T. The magnetization **M** is specified as 113.8 kA/m [22]. As shown in Figure 10, as the applied magnetic field strength increases, the field variables $\lambda_{1}$, ξ, $\sigma_{1}^{*}$, and $z^{*}$ at the center of the HMEM increase gradually. When $t^{*}$ exceeds 12, the variations in these field variables almost remain unchanged and finally evolve into a stable state.

**Figure 11:**
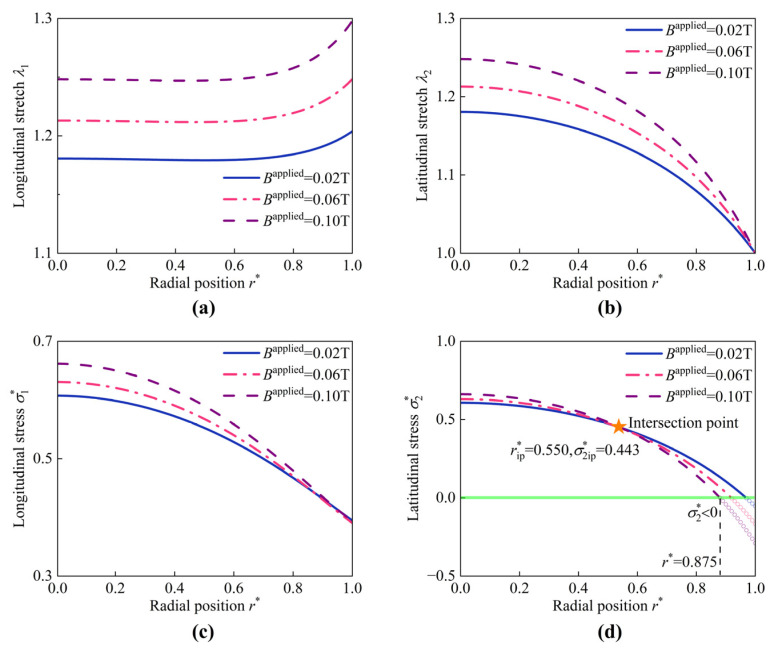
The hyperelastic deformations of the HMEM under different values of $B^{applied}$ ($p^{*}$ = 0.6). (**a**) The longitudinal stretch $\lambda_{1}$, (**b**) latitudinal stretch $\lambda_{2}$, (**c**) longitudinal stress $\sigma_{1}^{*}$, and (**d**) latitudinal stress $\sigma_{2}^{*}$.

**Figure 12:**
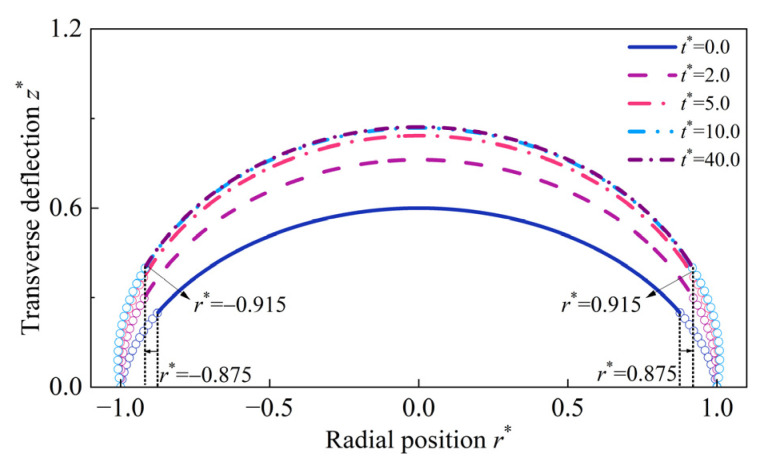
The evolution shape of the HMEM under ($p^{*}$ = 0.6, $B^{applied}$ = 0.1 T).

The hyperelastic deformations of the HMEM for different values of $B^{applied}$ are illustrated in Figure 11. The curves of the longitudinal stretch $\lambda_{1}$ are non-monotonic, initially decreasing a little bit and then increasing. $\lambda_{2}$ gradually decreases and keeps 1.0 at the boundary $r^{*}$ = 1.0. $\sigma_{1}^{*}$ and $\sigma_{2}^{*}$ decreases monotonically along the radial direction. It can be seen that as $B^{applied}$ increases from 0.02 T to 0.1 T, the distribution curves of $\sigma_{2}^{*}$ exhibit an intersection point (abbreviated as subscript ‘ip’) as shown in Figure 11d, e.g., ($r_{ip}^{*}$ = 0.550,$\sigma_{2ip}^{*}$ = 0.443). On the left part $0\leq r^{*}\leq r_{ip}^{*}$, $\sigma_{2}^{*}$ decreases with increasing $B^{applied}$, whereas on the right part $r_{ip}^{*}\leq r^{*}\leq 1$, the trend is reversed. Furthermore, it is observed that $\sigma_{2}^{*}$ is less than 0 at the region $r^{*}$ = 0.875 to $r^{*}$ = 1.0, indicating the transformation from tensile stresses to compressive stress. The occurrence of compressive stress may lead to wrinkling. A similar phenomenon was reported in the work [60]. Despite the HMEM being in a ‘stretched’ state ($\lambda_{1}>1$ and $\lambda_{2}>1$) under magneto-pneumatic coupling, wrinkles appear because the total Cauchy stresses are compressive. The evolution shape of the HMEM under ($p^{*}$ = 0.6,$B^{applied}$ = 0.1 T) is depicted in Figure 12. Initially, at $t^{*}$ = 0, the HMEM undergoes hyperelastic deformation, with wrinkles appearing at $r^{*}$ = 0.875. As the evolution time $t^{*}$ progresses to 40, the position of the wrinkling moves to $r^{*}$ = 0.915.

**Figure 13:**
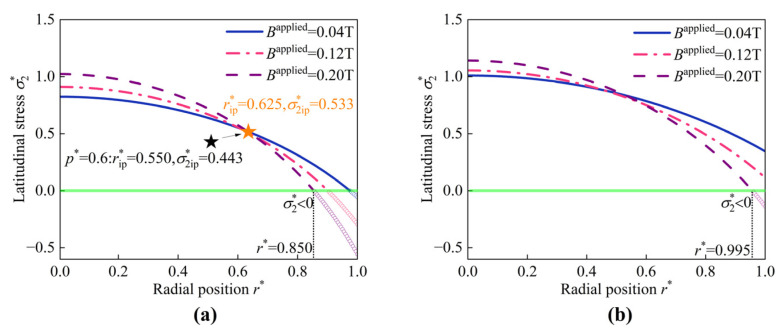
(**a**) The latitudinal stress $\sigma_{2}^{*}$ under $p^{*}$ = 0.8. (**b**) The latitudinal stress $\sigma_{2}^{*}$ under $p^{*}$= 0.8 and *k* = 1.1.

As shown in Figure 13a, when $p^{*}$ increases from 0.6 to 0.8, the position of the intersection point moves rightwards, which means ($r_{ip}^{*}$ = 0.550, $\sigma_{2ip}^{*}$ = 0.443) shifts to ($r_{ip}^{*}$ = 0.625, $\sigma_{2ip}^{*}$ = 0.533). This phenomenon indicates that pressure has an impact on the position of the intersection point. By comparing the results in Figure 13a,b, it is observed that as pre-stretch *k* increases from 1.0 to 1.1, $\sigma_{2}^{*}$ gradually becomes greater than 0, indicating that the wrinkle disappears. This demonstration reveals that the pre-stretch *k* is capable of suppressing the wrinkle.

**Figure 14:**
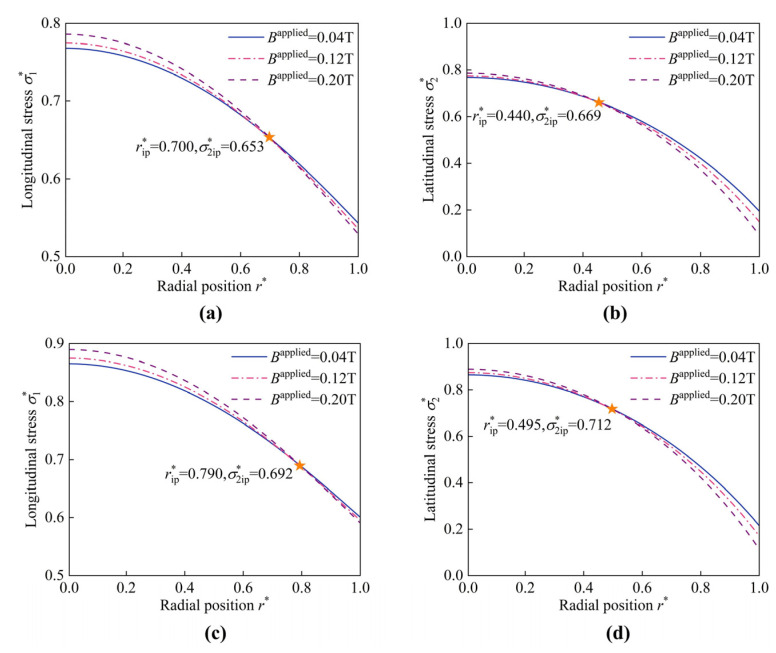
The schematic represents (**a**) The longitudinal stress $\sigma_{1}^{*}$ and (**b**) the latitudinal stress $\sigma_{2}^{*}$ under $p^{*}$ = 0.8 and *G* = 300 kPa; (**c**) The longitudinal stress $\sigma_{1}^{*}$ and (**d**) the latitudinal stress $\sigma_{2}^{*}$ under $p^{*}$ = 0.8 and *k* = 1.1, *G* = 300 kPa.

Additionally, wrinkles can be eliminated by increasing shear modulus G, as illustrated in Figure 14. The intersection points are observed for the distribution curves of both $\sigma_{1}^{*}$ and $\sigma_{2}^{*}$. With a pre-stretch *k* = 1.1, these intersection points shift to the right. Specifically, the position of the intersection point of $\sigma_{1}^{*}$, i.e., ($r_{ip}^{*}$ = 0.700, $\sigma_{2ip}^{*}$ = 0.653) for *k* = 1 (see Figure 14a) shifts to ($r_{ip}^{*}$ = 0.790, $\sigma_{2ip}^{*}$ = 0.692) (see Figure 14c). Similarly, the intersection point ($r_{ip}^{*}$ = 0.440, $\sigma_{2ip}^{*}$ = 0.669) in $\sigma_{2}^{*}$ (see Figure 14b) shifts to ($r_{ip}^{*}$ = 0.495, $\sigma_{2ip}^{*}$ = 0.712) (see Figure 14d). Both the pressure and the pre-stretch display an effective influence on the positions of the intersection points.

**Figure 15:**
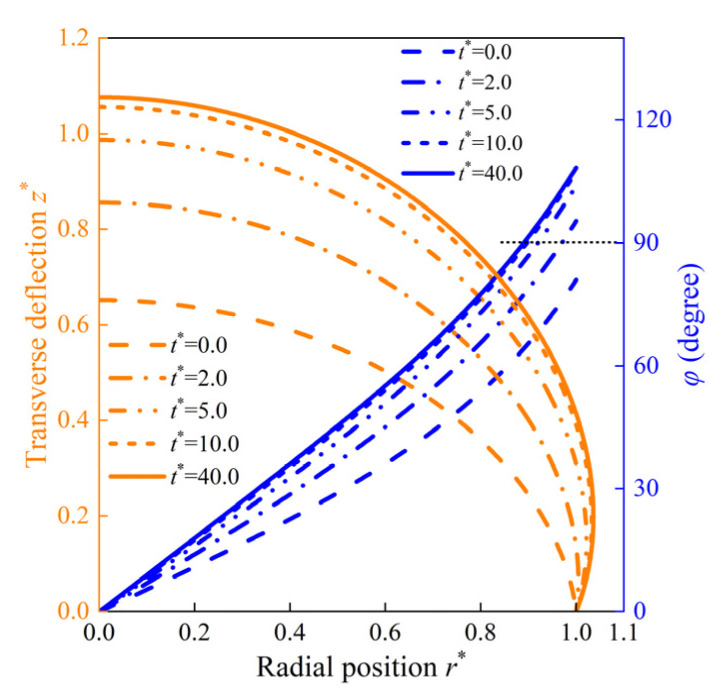
The schematic represents the transverse deflection $z^{*}$ and the corresponding angle under ($p^{*}$ = 0.8,$B^{applied}$ = 0.1 T) at $t^{*}$ = 0, 2, 5, 10, and 40.

The shape of the HMEM may evolve beyond the boundary positions ($r^{*}>1$), and the angles at the corresponding positions are larger than 90 degrees, as depicted in Figure 15. As $t^{*}$ goes from 0 to 10, both the deformation and angles increase gradually. When $t^{*}$ progresses from 10 to 40, the changes in $z^{*}$ and φ are very small, indicating that the HMEM approaches a stable state.

**Figure 16:**
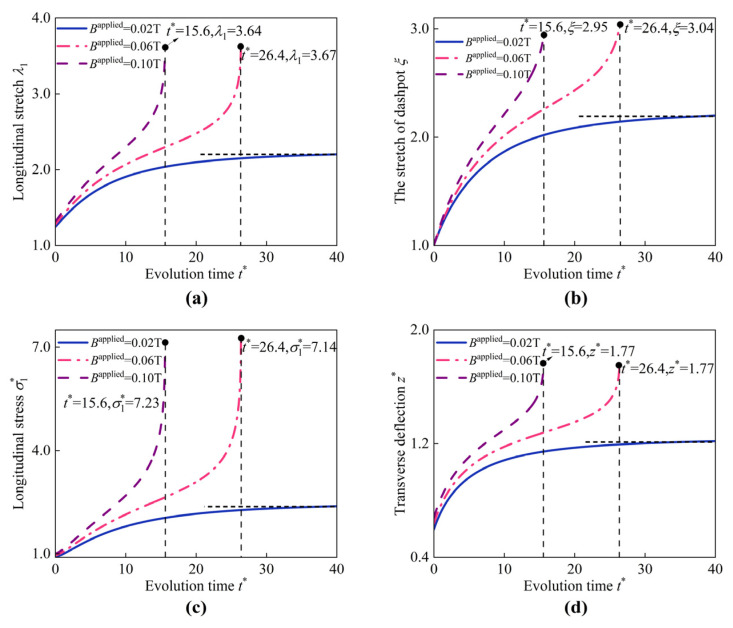
Evolution of (**a**) longitudinal stretch $\lambda_{1}$, (**b**) the stretch of dashpot ξ, (**c**) longitudinal stress $\sigma_{1}^{*}$, and (**d**) transverse deflection $z^{*}$ at the center of the HMEM under different values of $B^{applied}$ ($p^{*}=0.9$).

Here, the influence of the applied magnetic field on the instability of the HMEM is considered, as depicted in Figure 16. It is well-established that the HMEM evolves into a stable state when inflated by pure pressure $p^{*}$ = 0.9 ($B^{applied}$ = 0 T). As the applied magnetic field $B^{applied}$ increases from 0.02 T to 0.1 T, the HMEM cannot evolve into a stable state. For example, when $B^{applied}$ = 0.06 and 0.1 T, the HMEM evolves into instability at the time $t^{*}$ = 26.4 and 15.6, respectively. As $B^{applied}$ increases, the instability time of the HMEM advances, speeding up the HMEM’s evolution toward an unstable state.

**Figure 17:**
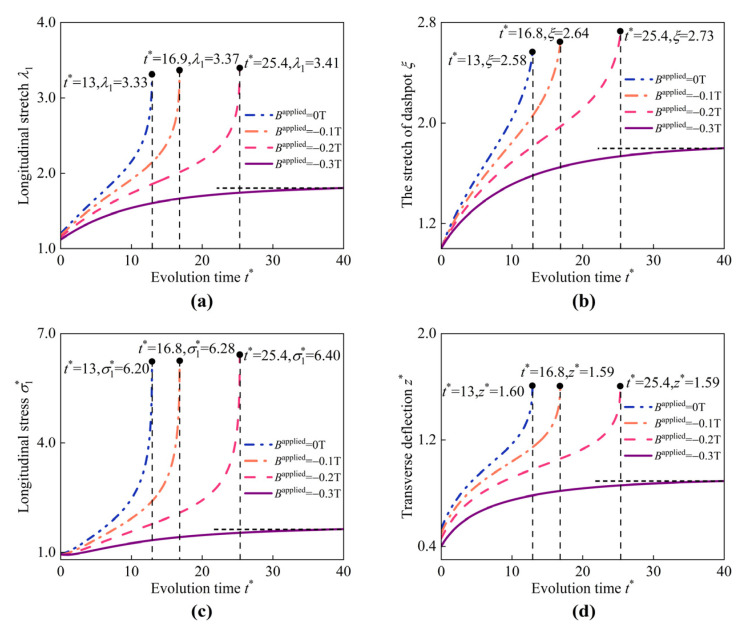
Evolution of (**a**) longitudinal stretch $\lambda_{1}$, (**b**) the stretch of dashpot ξ, (**c**) longitudinal stress $\sigma_{1}^{*}$, and (**d**) transverse deflection $z^{*}$ at the center of the HMEM under different values of $B^{applied}$ ($p^{*}$ = 1.0).

Referring to the results in Figure 8, under pure pressure $p^{*}$ = 1.0, the HMEM evolves into an unstable state at $t^{*}$ = 13. Building upon this conclusion, the influence of the reversed magnetic field on the instability is studied. As depicted in Figure 17, it can be seen that the HMEM can evolve into a stable state under $p^{*}$ = 1.0 and $B^{applied}=-0.3T$. When $B^{applied}=-0.2T$, the instability occurs at $t^{*}$ = 25.4, while under $B^{applied}=-0.1T$, the instability appears at $t^{*}$ = 16.8. The results indicate that increasing reversed magnetic field can suppress the instability in HMEM.

**Figure 18:**
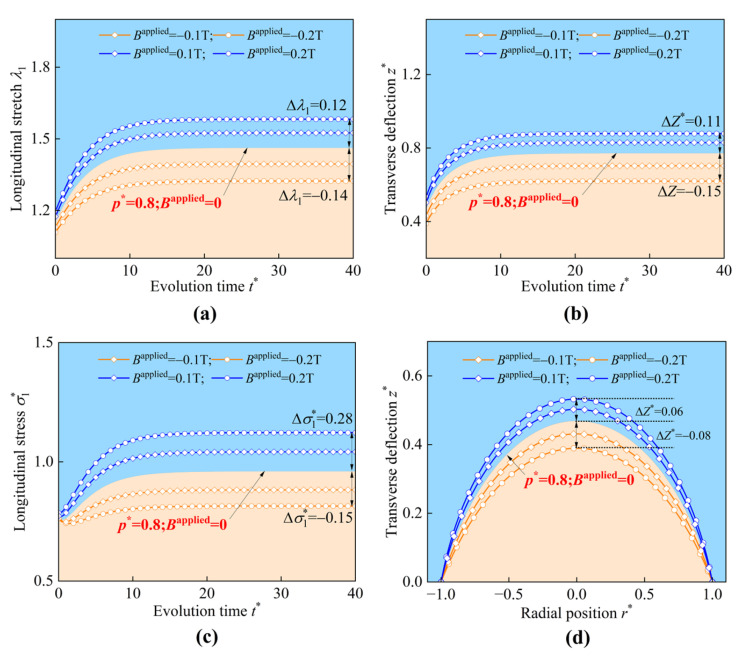
The schematic represents (**a**) the longitudinal stretch $\lambda_{1}$, (**b**) the transverse deflection $z^{*}$, (**c**) the longitudinal stress $\sigma_{1}^{*}$ at the center of the HMEM, and (**d**) The shape of the HMEM under different values of $B^{applied}$ ($p^{*}$ = 0.8).

In Figure 18, the boundary lines of the two regions represent the corresponding physical quantities with no external magnetic field ($B^{applied}$ = 0). The curves in the blue region and orange region display the magnetomechanical behaviors under different magneto-pneumatic coupling. When the applied magnetic fields align with **k**, the inflation of the HMEM is enhanced, whereas the applied magnetic fields are in the opposite direction of **k**; the inflation of the HMEM is then suppressed.

**Figure 19:**
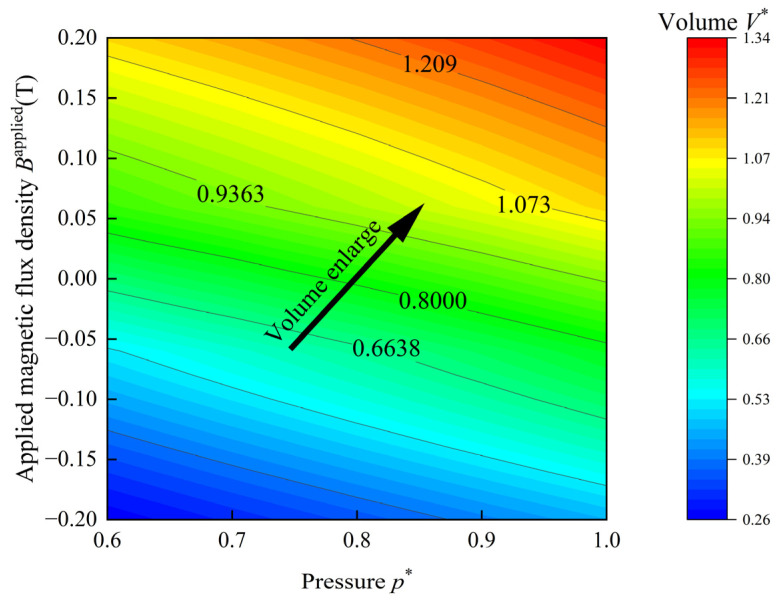
Contours of the volume of the HMEM subjected to magneto-pneumatic coupling loadings.

The contours of the volume of the HMEM under different values of $p^{*}$ and $B^{applied}$ are depicted in Figure 19. The volume enlarges with the increase in $p^{*}$ and $B^{applied}$ (as indicated by the black arrow). When $B^{applied}$ is greater than 0, the applied magnetic field enhances the volume enlargement of the HMEM. For $B^{applied}<0$, the effect is the opposite.

## 5. Conclusions

We have developed a theoretical model for hard-magnetic elastomer membranes which accounts for:

1. Hyperelastic deformations of the HMEM.
2. Viscoelasticity and instability of the HMEM.

By solving the governing equations through the shooting method and the improved Euler method, field variables of the HMEM can be obtained. These numerical results have been validated by comparing them with finite element simulation by COMSOL under different magneto-pneumatic coupling. Furthermore, by considering different values of the magneto-pneumatic coupling, magnetomechanical behaviors of the HMEM are systematically investigated. The main conclusions are as follows: (i) When subjected to pure pressure exceeding 0.9, the HMEM gradually evolves into an unstable state. (ii) Under magneto-pneumatic coupling, wrinkles occur in the HMEM when $\sigma_{2}^{*}$ is less than 0. These wrinkles can be eliminated by applying pre-stretch or increasing shear modulus. (iii) The intersection points of the distribution curves for $\sigma_{1}^{*}$ and $\sigma_{2}^{*}$ are identified, which is only controlled by pre-stretch and pressure. (iv) The instability of the HMEM is significantly influenced by applied magnetic fields, indicating that applying a magnetic field aligned with the positive (negative) *z*-axis speeds up (suppresses) the instability in HMEM. For example, when $p^{*}$ and $B^{applied}$ equal 1.0 and −0.3 T, respectively, the HMEM no longer experiences instability. The presented results provide valuable insights in designing and manufacturing high-performance hard-magnetic membrane structures and actuators under the pre-stretch and magneto-pneumatic coupling.

## Data Availability

The original contributions presented in the study are included in the article, further inquiries can be directed to the corresponding authors.

## Appendix A

To simplify the solving process, the following non-dimensional quantities are introduced as follows:

(A1) $$\begin{array}{l} s_{1}^{*}=\frac{s_{1}}{G},s_{2}^{*}=\frac{s_{2}}{G},r^{*}=\frac{R}{R_{0}},z^{*}=\frac{z}{R_{0}},G_{\alpha }^{*}=\frac{G_{\alpha }}{G};t^{*}=\frac{t}{t_{v}}\\ G_{\beta }^{*}=\frac{G_{\beta }}{G},p^{*}=\frac{pR}{GH},B^{*}=\frac{B}{\sqrt{\mu_{0}G}} \end{array}$$

The system of the non-dimensional first-order ordinary differential equations is

(A2) $$\frac{\partial r^{*}}{\partial R^{*}}=\lambda_{1}\cos\varphi$$

(A3) $$\frac{d\varphi }{dR^{*}}=-\frac{s_{2}^{*}}{s_{1}^{*}}\frac{sin\varphi }{R}+\frac{\lambda_{1}\lambda_{2}p^{*}}{s_{1}^{*}}$$

(A4) $$\frac{d\lambda_{1}}{dR^{*}}=\frac{1}{f_{1}^{*}}\left[(\frac{s_{2}^{*}cos\varphi -s_{1}^{*}}{R})-f_{2}^{*}(\frac{\lambda_{1}cos\varphi -\lambda_{2}}{R^{*}})+f_{3}^{*}\frac{d\xi_{1}}{dR^{*}}+f_{4}^{*}\frac{d\xi_{2}}{dR^{*}}\right]$$

where

(A5) $$\begin{array}{l} f_{1}^{*}=G_{\alpha }^{*}+3G_{\alpha }^{*}\lambda_{1}^{-4}\lambda_{2}^{-2}+G_{\beta }^{*}\xi_{1}^{-2}+3G_{\beta }^{*}\xi_{1}^{2}\xi_{2}^{2}\lambda_{1}^{-4}\lambda_{2}^{-2}+3\lambda_{2}^{-2}\lambda_{1}^{-4}B^{{*}^{2}}\\ f_{2}^{*}=2G_{\alpha }^{*}\lambda_{1}^{-3}\lambda_{2}^{-3}+2G_{\beta }^{*}\xi_{1}^{2}\xi_{2}^{2}\lambda_{1}^{-3}\lambda_{2}^{-3}+2\lambda_{1}^{-3}\lambda_{2}^{-3}B^{{*}^{2}}\\ f_{3}^{*}=2G_{\beta }^{*}(\lambda_{1}^{-3}\lambda_{2}^{-2}\xi_{1}\xi_{2}^{2}+\lambda_{1}\xi_{1}^{-3})\\ f_{4}^{*}=2G_{\beta }^{*}\lambda_{1}^{-3}\lambda_{2}^{-2}\xi_{1}^{2}\xi_{2} \end{array}$$

(A6) $$\frac{\partial z^{*}}{\partial R^{*}}=-\lambda_{1}sin\varphi$$

The non-dimensional form of the nominal stresses $s_{1}$ and $s_{2}$ are

(A7) $$s_{1}^{*}=G_{\alpha }^{*}(\lambda_{1}-\lambda_{1}^{-3}\lambda_{2}^{-2})+G_{\beta }^{*}(\lambda_{1}\xi_{1}^{-2}-\xi_{1}^{2}\xi_{2}^{2}\lambda_{1}^{-3}\lambda_{2}^{-2})-\lambda_{1}^{-3}\lambda_{2}^{-2}B^{{*}^{2}}$$

(A8) $$s_{2}^{*}=G_{\alpha }^{*}(\lambda_{2}-\lambda_{1}^{-2}\lambda_{2}^{-3})+G_{\beta }^{*}(\lambda_{2}\xi_{2}^{-2}-\xi_{1}^{2}\xi_{2}^{2}\lambda_{1}^{-2}\lambda_{2}^{-3})-\lambda_{1}^{-2}\lambda_{2}^{-3}B^{{*}^{2}}$$
